# Pinocembrin protects against β-amyloid-induced toxicity in neurons through inhibiting receptor for advanced glycation end products (RAGE)-independent signaling pathways and regulating mitochondrion-mediated apoptosis

**DOI:** 10.1186/1741-7015-10-105

**Published:** 2012-09-18

**Authors:** Rui Liu, Cai-xia Wu, Dan Zhou, Fan Yang, Shuo Tian, Li Zhang, Tian-tai Zhang, Guan-hua Du

**Affiliations:** 1 National Center of Pharmacological Screening, Institute of Materia Medica, Chinese Academy of Medical Sciences & Peking Union Medical College, Beijing 100050, P R China; 2 State Key Laboratory of Bioactive Substance and Function of Natural Medicines, Institute of Materia Medica, Chinese Academy of Medical Sciences and Peking Union Medical College, Beijing 100050, P R China; 3 Shenyang Pharmaceutical University, Shenyang, 110016, P R China

**Keywords:** Alzheimer's disease, amyloid-β peptide, apoptosis, pinocembrin, receptor for advanced glycation end products

## Abstract

**Background:**

It is known that amyloid-β peptide (Aβ) plays a pivotal role in the pathogenesis of Alzheimer's disease (AD). Interaction between Aβ and the receptor for advanced glycation end products (RAGE) has been implicated in neuronal degeneration associated with this disease. Pinocembrin, a flavonoid abundant in propolis, has been reported to possess numerous biological activities beneficial to health. Our previous studies have demonstrated that pinocembrin has neuroprotective effects on ischemic and vascular dementia in animal models. It has been approved by the State Food and Drug Administration of China for clinical use in stroke patients. Against this background, we investigated the effects of pinocembrin on cognitive function and neuronal protection against Aβ-induced toxicity and explored its potential mechanism.

**Methods:**

Mice received an intracerebroventricular fusion of Aβ_25-35_. Pinocembrin was administrated orally at 20 mg/kg/day and 40 mg/kg/day for 8 days. Behavioral performance, cerebral cortex neuropil ultrastructure, neuronal degeneration and RAGE expression were assessed. Further, a RAGE-overexpressing cell model and an AD cell model were used for investigating the mechanisms of pinocembrin. The mechanisms underlying the efficacy of pinocembrin were conducted on target action, mitochondrial function and potential signal transduction using fluorescence-based multiparametric technologies on a high-content analysis platform.

**Results:**

Our results showed that oral administration of pinocembrin improved cognitive function, preserved the ultrastructural neuropil and decreased neurodegeneration of the cerebral cortex in Aβ_25-35_-treated mice. Pinocembrin did not have a significant effect on inhibiting Aβ_1-42 _production and scavenging intracellular reactive oxygen species (ROS). However, pinocembrin significantly inhibited the upregulation of RAGE transcripts and protein expression both *in vivo *and *in vitro*, and also markedly depressed the activation of p38 mitogen-activated protein kinase (MAPK)-MAPKAP kinase-2 (MK2)-heat shock protein 27 (HSP27) and stress-activated protein kinase (SAPK)/c-Jun N-terminal kinase (JNK)-c-Jun pathways and the downstream nuclear factor κB (NFκB) inflammatory response subsequent to Aβ-RAGE interaction. In addition, pinocembrin significantly alleviated mitochondrial dysfunction through improving mitochondrial membrane potential and inhibiting mitochondrial oxidative stress, and regulated mitochondrion-mediated apoptosis by restoration of B cell lymphoma 2 (Bcl-2) and cytochrome *c *and inactivation of caspase 3 and caspase 9.

**Conclusions:**

Pinocembrin was shown to infer cognitive improvement and neuronal protection in AD models. The mechanisms of action of the compound were illustrated on RAGE-dependent transduction inhibition and mitochondrion protection. It appears to be a promising candidate for the prevention and therapy of AD.

## Background

Alzheimer's disease (AD) is a progressive neurodegenerative disease characterized by the presence of senile plaques, intracellular neurofibrillary tangles and neuronal loss [1]. Amyloid-β peptide (Aβ) is found in extracellular senile plaque cores and is recognized as one of the vital neuropathological hallmarks of AD. Although the exact mechanism of Aβ-induced cell damage is unclear, multiple molecular pathways resulting in cell death are involved in vulnerable neuronal populations [2,3]. Surrounding the amyloid plaques, there is an apparent chronic progressive inflammatory response in neuronal cells [4]. Cytoplasmic blebbing, mitochondrial calcium dyshomeostasis, cytochrome *c *release, chromatin condensation, nuclear damage, and DNA fragmentation can also be activated locally following exposure to Aβ [5]. Increased intracellular Aβ levels could further facilitate opening of the mitochondrial permeability transition pores [6]. This indicates that Aβ can directly disrupt mitochondrial function, reduce energy metabolism and contribute to the mitochondrion-dependent apoptosis.

Aβ is a pleiotropic peptide and is capable of binding to the receptors at several different membrane locations [7]. The receptor for advanced glycation end products (RAGE), a multiligand receptor of the immunoglobulin superfamily of cell surface molecules [8-10], possesses a cell surface binding site for Aβ peptides [7] and is expressed at higher levels when stimulated by excessive amounts of Aβ [11,12]. RAGE has been extensively studied for its role in migration and differentiation of neuronal cells during development, perturbation of neuronal cells by Aβ and for its role in the inflammatory response [11,13-15]. The wild-type RAGE transgene targeted to neurons in the transgenic AD mouse model that expressed mutant human amyloid precursor protein (APP) has been shown to accelerate Aβ-mediated neuronal perturbation [16,17]. In the same way, administration of Aβ has been shown to be cerebrotoxic, and RAGE was also robustly expressed throughout the adult rat brain in neurons and glia in ischemic pathology [18]. As a consequence of Aβ-RAGE interaction, activation of p38 mitogen-activated protein kinases (p38MAPK), stress-activated protein kinase or c-Jun N-terminal kinase (SAPK/JNK), and nuclear factor κB (NFκB) signaling transduction was seen in synaptic failure of sporadic AD cybrids [15]. Therefore, Aβ-RAGE is known to be a key mediator of neuronal damage in AD, as well as a contributor to stroke pathology through dependent upregulation of inflammatory cytokines and NFκB [19].

**Figure 1:**
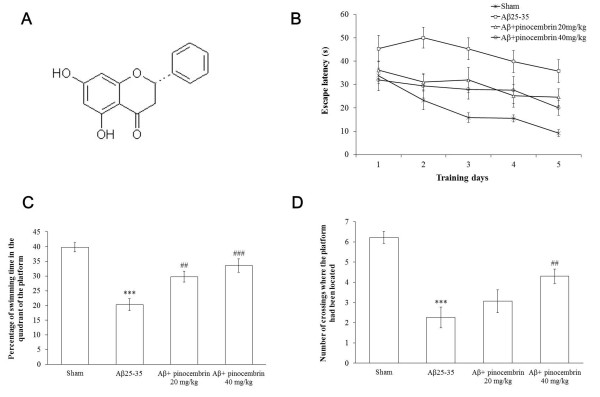
**Chemical structure of pinocembrin and the effect of pinocembrin on the behavior of amyloid-β peptide (Aβ)_25-35_-treated mice in Morris water maze (MWM)**. **(A) **Structure of pinocembrin. **(B) **Comparison of latency to platform during 5 days of training in MWM. Acquisition training was initiated on the third day of pinocembrin oral administration (day 1 for MWM test). Daily training consisted of four trials in which the mouse was placed in the water from four random starting positions (north, east, south, and west) and the escape latency onto the platform was recorded. Data are presented as the mean ± SEM. In all 12 mice were tested per group. **(C) **Pinocembrin increased the percentage of time the mouse stayed in the target quadrant in the probe test. ***P < 0.01 vs sham, ^##^*P *< 0.01, ^###^*P *< 0.001 vs Aβ_25-35_. Data are presented as the mean ± SEM, n = 12 mice per group. **(D) **Pinocembrin increased the numbers of crossings where the platform had been located in the probe test. ***P < 0.01 vs sham, ^##^*P *< 0.01 vs Aβ_25-35_. Data are presented as the mean ± SEM, n = 12 mice per group.

Pinocembrin (5,7-dihydroxyflavanone; Figure 1A) is a natural flavonoid found at high concentration in propolis. It has been extracted as a pure compound from propolis, and subsequently pinocembrin was synthesized and approved by the State Food and Drug Administration (SFDA) of China for stroke clinical trials in 2008. This compound is well metabolized and can circulate in the body after oral administration [20]. In particular, it is able to pass through the blood-brain barrier (BBB) in a passive transport process partly conducted by P-glycoprotein [21]. Our previous studies have showed that pinocembrin reduced glutamate-induced SH-SY5Y cell injury, protected primary cortical neurons against oxygen-glucose deprivation/reoxygenation injury [22,23], and reduced the area of cerebral infarct [22,24,25]. Moreover, pinocembrin had potent neuroprotective effects by improving mitochondrial function [22,26], decreasing oxidative damage [22-24], reducing neuronal apoptosis [22,23], and inhibiting inflammatory responses [22] in middle cerebral artery occlusion rat models. Recently, we found that pinocembrin alleviated learning and memory deficits in a vascular dementia rat model through mitochondrial protection [27], which suggests that pinocembrin has potential therapeutic effects for cognitive impairment.

Even though experimental evidence suggests that pinocembrin exerts neuroprotective effects and improves cognitive function, no pre-existing study has reported the exact molecular mechanism of this compound. Thus, as a part of our ongoing evaluating program to explore the potential mechanisms, we examined the effects of pinocembrin on improving cognitive impairment in mice induced by intracerebroventricular infusion of Aβ_25-35_. Furthermore, we investigated the mechanisms underlying the efficacy of the compound on target action, mitochondrial function and potential signal transduction in different cell models using fluorescence-based multiparametric technologies with a high-content analysis platform.

## Methods

### Animals and treatment

Pinocembrin (high performance liquid chromatography (HPLC) purity >98%) was synthesized by the Department of Medical Synthetic Chemistry, Institute of Materia Medica, Beijing, P R China. Aβ_25-35 _and Aβ_1-42 _were purchased from Sigma Chemical Co (St Louis, MO, USA). Aβ_25-35 _was dissolved in sterile saline (1 mM) and aggregated by incubation at 37°C for 7 days before use.

Male Kunming mice, 25 to 30 g, were provided by the Animal Breeding Center of the Chinese Academy of Medical Sciences. Mice were housed five per cage and acclimated to standard laboratory conditions (12 h light, 12 h dark cycle) with free access to mouse chow and water. The animal breeding and experiments were conducted in accordance with institutional guidelines and ethics and approved by the Laboratories Institutional Animal Care and Use Committee of Chinese Academy of Medical Sciences and Peking Union Medical College.

Mice were anesthetized with 50 mg/kg sodium pentobarbital (Sigma, St. Louis, MO, USA) and placed in a stereotaxic instrument (RWD Life Science, Shenzhen, China). The aggregated Aβ_25-35 _was injected into the right lateral ventricle with the following coordinates: -0.5 mm anterior/posterior, +1.0 mm medial/lateral and -2.5 mm dorsal/ventral from Bregma (10 nmol in 3 μl of saline per injection). Sham animals were injected in an identical manner with the same amount of sterile saline.

A total of 48 mice were used, allocated to one of four groups the day after sterile saline or Aβ_25-35 _injection: sham group, Aβ_25-35_-treated group, pinocembrin 20 mg/kg group, and pinocembrin 40 mg/kg group (12 mice in each group). Pinocembrin was dissolved in distilled water containing 20% hydroxypropyl-β-cyclodextrin (Sigma, St Louis, MO, USA) at a concentration of 10 mg/ml, and was administered by oral gavage once a day continuously for 8 days. The sham group and Aβ_25-35_-treated group received oral gavage in the same manner using distilled water containing 20% hydroxypropyl-β-cyclodextrin without pinocembrin. After behavioral testing was completed, half the mice in each group were anaesthetized and killed by decapitation. The brains were quickly dissected and then snap frozen in liquid nitrogen. The brains were stored at -80°C before analysis. The other mice in each group were anaesthetized and perfused with 0.9% saline (Beijing Chemical Works, Beijing, China) and 4% paraformaldehyde (pH 7.40~7.50; Beijing Chemical Works, Beijing, China) from the left ventricle of heart. The division of mice into treatment groups and the selection of mice to be killed within each group were both performed randomly.

### Morris water maze (MWM) performance

The MWM task was used to evaluate the learning and memory changes in mice [28]. Briefly, maze training began on the third day of pinocembrin administration. In each trial, the time required to escape onto the hidden platform was recorded as escape latency. Mice were trained for five consecutive days. On the ninth day of pinocembrin treatment, a single probe trial was conducted. The time the mouse stayed in the platform quadrant and the crossings where the platform had been located were recorded. The treatments were continued during the water maze task.

### Transmission electron microscopy (TEM) assay

For the TEM assay, mice were anaesthetized with an intraperitoneal injection of 45 mg/kg sodium pentobarbital (Sigma, St. Louis, MO, USA). The heart was exposed and the left ventricle was perfused with 0.9% saline, followed by perfusion with 4% paraformaldehyde (Beijing Chemical Works, Beijing, China). At the end of brain perfusion, the temporal cerebral cortices were isolated carefully and placed in fixative [approximately 20 ml of 2.5% glutaraldehyde (Merck, Darmstadt, Germany) and 2.0% paraformaldehyde (Beijing Chemical Works, Beijing, China) in 0.15 M cacodylate buffer (Merck, Darmstadt, Germany)] overnight. Then, the samples were post-fixed in 1% osmium tetroxide (Sigma, St. Louis, MO, USA), stained in 2% uranyl acetate (Sigma, St. Louis, MO, USA), dehydrated in ethanol (Beijing Chemical Works, Beijing, China) and acetone (Beijing Chemical Works, Beijing, China), and embedded in epoxy resin (Beijing Zhongjingkeyi Technology, Beijing, China). Ultrathin sections (60 nm thick) were obtained of selected blocs of the samples. The ultrathin sections were mounted on copper grids (200 mesh; Beijing Zhongjingkeyi Technology, Beijing, China) and double-contrasted with uranyl acetate and lead citrate (Sigma, St. Louis, MO, USA) for examination in a LEO 906 transmission electron microscope (Zeiss, Oberkochen, Germany) operated at 60 kV.

### Fluoro-Jade B staining assay

Fluoro-Jade B (FJB; Histochem, Jefferson, AR, USA) was used to determine neuron degeneration induced by Aβ_25-35 _in the cerebral cortex as described previously [29,30]. Briefly, the slides were first immersed in 100% ethanol (Beijing Chemical Works, Beijing, China), followed by 70% ethanol and 30% ethanol. These slides were then oxidized by soaking in 0.06% potassium permanganate (Sigma, St Louis, MO, USA), and transferred to a 0.0004% FJB for 30 min. After washing, the slides were air dried, cleared in xylene (Beijing Chemical Works, Beijing, China), and coverslipped with a mounting medium for histological detection. The slices were examined using an Olympus IX71 fluorescent microscope (Olympus, Tokyo, Japan) with blue excitation light and a barrier filter. Neurons undergoing degeneration showed bright fluorescence in comparison to the background. The number of FJB-positive neurons was counted in a section. In the cerebral cortex regions the number of FJB-positive neurons was calibrated as the number of neurons in 1 mm^2^. Cell counts were obtained by averaging the counts from 10 sections taken from each mouse.

### RNA analysis and western blot assay

The expression of RAGE in cerebral cortex was determined by quantitative real-time polymerase chain reaction (PCR) and western blot. Total RNA was extracted from part of the tissues using TRIzol reagents (Invitrogen, Carlsbad, CA, USA) and was processed directly to produce cDNA using TaqMan reverse transcription reagents kit (Applied Biosystems, Foster City, CA, USA). Quantitative real-time PCR was performed on the ABI Prism 7500 Sequence Detection System (Applied Biosystems, Foster City, CA, USA) with Power SYBR Green PCR Master Mix (Applied Biosystems, Foster City, CA, USA). Mouse RAGE primer and probe consist of forward primer, 5'-ACAGGCGAGGGAAGGAGGTCAAGT-3'; reverse primer, 5'-TGGGCAGAGATGGCACAGGTCA-3') (Genbank: L33412). β-Actin was used as a control. Data were calculated using the 2^-ΔΔ*Ct *^method and are expressed as fold increase over the indicated controls.

Other samples of the tissues were used to determine protein expression of RAGE. Proteins were extracted from the pellets by using lysis buffer [50 mM Tris-HCl (pH 7.4; Sigma, St Louis, MO, USA), 20 mM ethylenediaminetetra-acetic acid (EDTA; Beijing Chemical Works, Beijing, China), 0.1% sodium dodecyl sulfate (SDS; Sigma, St Louis, MO, USA), 100 mM NaCl (Beijing Chemical Works, Beijing, China), 1% NP-40 (Sigma, St Louis, MO, USA), 0.5% sodium deoxycholate (Sigma, St Louis, MO, USA), 50 mM sodium fluoride (Sigma, St Louis, MO, USA), 1 mM sodium orthovanadate (Sigma, St Louis, MO, USA), 1 mM phenylmethanesulfonylfluoride (PMSF; Sigma, St Louis, MO, USA), 2 mM sodium pyrophosphate (Sigma, St Louis, MO, USA), 1 μg/ml pepstatin A (Sigma, St Louis, MO, USA), 100 μg/ml leupeptin (Sigma, St Louis, MO, USA) and one protease inhibitor cocktail tablet (1/50 ml; Roche Molecular Biochemicals, Indianapolis, IN, USA)]. Samples were boiled for 3 min before loading onto SDS-polyacrylamide gel. After electrophoresis, the gel was electroblotted onto polyvinylidene difluoride membranes. Membranes were blocked in Tris-buffered saline (TBS; Sigma, St Louis, MO, USA) with 1% Tween-20 (TBST; Sigma, St Louis, MO, USA) and 5% non-fat dry milk (Sigma, St Louis, MO, USA), and then incubated with anti-RAGE (1:800, Cell Signaling Technology, Bervely, MA, USA ) overnight at 4°C. Membranes were washed several times with TBST prior to incubation with Horseradish peroxidase (HRP)-conjugated secondary antibody (1:1,000, ZSGB-Bio, Beijing, China) for 45 min at room temperature. After subsequent washes in TBST, the protein bands were visualized using an ECL™ detection kit (GE Healthcare; Piscataway, NJ, USA) and exposure to X-ray films. Relative optical densities and areas of bands were quantified using the Image J densitometry software (version 1.6, National Institutes of Health, Bethesda, MD, USA). The densitometric plots of the results were normalized to the intensity of the actin band.

### Cell cultures, transfections and treatments

U2OS cells were firstly applied to be stably transduced with a reporter plasmid (pEGFP-N1) encoding a strong response region of human RAGE promoter [31] as a RAGE-overexpressing cell model, and then coincubated with pinocembrin in order to specifically dissect whether there is an effect on RAGE expression U2OS cells were grown in McCoy's 5A medium (Invitrogen, Carlsbad, CA, USA) supplemented with 10% fetal calf serum (FBS; Gibco/Invitrogen, Grand Island, NY, USA) at 37°C in humidified 5% CO_2 _air. The enhanced green fluorescent protein (EGFP)-based plasmid encoding human RAGE promoter was transfected into U2OS cells and the stably expressing cells were selected by G418 resistance (Invitrogen, Carlsbad, CA, USA). The RAGE-overexpressing cell model was established by using Aβ_1-42 _to induce RAGE transcription and trigger RAGE overexpression. Cells were treated with 50 nM Aβ_1-42 _for 24 h, or coincubated Aβ_1-42 _with 1.0 μM, 3.0 μM, or 10.0 μM pinocembrin for 24 h. The cells were then randomly divided into five groups: (1) RAGE control group; (2) RAGE group in the presence of 50 nM Aβ_1-42 _for 24 h; RAGE group in the presence of 50 nM Aβ_1-42 _with pinocembrin treatment at (3) 1.0 μM, (4) 3.0 μM, and (5) 10 μM for 24 h. After the treatment, the cells were imaged and analyzed by a fluorescence-based Cellomics ArrayScan high-content screening (HCS) Reader (Thermo Fisher Scientific Cellomics, Pittsburgh, PA, USA) with the Morphology Explorer BioApplication. The optical fields were scanned with a 20 × objective lens to obtain a minimum of 1,000 cells per well. The EGFP images and fluorescent intensity were acquired using a 485/20 nm excitation and 535/50 nm emission filters with a 600 ms exposure time. The level of RAGE overexpression was illustrated by the value of mean average fluorescent intensity (Mean_AvgInten). Percentage of RAGE expression inhibition was calculated using the following formula: 100 × (Aβ_25-35_-treated Mean_AvgInten - pinocembrin-treated Mean_AvgInten) ÷ Aβ_25-35_-treated Mean_AvgInten.

Human neuroblastoma SH-SY5Y cells overexpressing the Swedish mutant form of human APP (abbreviated to 'APPsw cells') were established as an AD cell model by using copper to trigger the neurotoxicity of Aβ. In this model, Aβ was overproduced in the cell line but had no toxicity during cultural process in the absence of Cu^2+^. In contrast, Aβ-mediated neurotoxicity was shown in the presence of Cu^2+^. This is an *in vitro *cell model for inducing Aβ-mediated neurotoxicity in which copper acts as a stimulator for Aβ when supplemented in culture medium.

Cells were treated with 300 μM copper (copper sulfate, Sigma, St Louis, MO, USA) for 24 h, and then given fresh medium containing or not 1.0 μM, 3.0 μM, and 10.0 μM pinocembrin to incubate 24 h. Cells were randomly divided into five groups: (1) APPsw group; (2) APPsw cells in the presence of 300 μM copper; (3) APPsw cells in the presence of 300 μM copper with pinocembrin treatment at 1.0 μM; (4) 3.0 μM; (5) 10.0 μM. Subsequently, the cells and medium were collected for detecting the effects of pinocembrin and the potential signaling pathway.

### Cell viability assay

The MTS (3-(4,5-dimethylthiazol-2-yl)-5-(3-carboxymethoxyphenyl)- 2-(4-sulfophenyl)-2H-tetrazolium, inner salt) assay was used for evaluating APPsw cell viability. After being treated with pinocembrin plus copper as described above, the medium was discarded and replaced with 100 μl of MTS solution (Promega, Madison, WI, USA) according to the manufacturer's protocol. After incubation at 37°C for 1 h, absorbance at 490 nm was measured on a SpectraMax Plus microplate reader (Molecular Devices, Sunnyvale, CA, USA).

### Measurements of intracellular reactive oxygen species (ROS)

ROS in APPsw cells were measured based on the oxidation of 2',7'-dihydrodichlorofluorescein diacetate (DCFH-DA; Sigma, St Louis, MO, USA) to 2',7'-dichlorofluorescein (DCF) [32]. DCF fluorescence intensity was detected and analyzed by a Cellomics ArrayScan V^TI ^HCS Reader (Thermo Fisher Scientific Cellomics, Pittsburgh, PA, USA) with the Morphology Explorer BioApplication. The cell images were acquired using a 485/20 nm excitation and 535/50 nm emission filters with a 300 ms exposure time. The level of intracellular ROS was illustrated by the value of mean average fluorescent intensity (Mean_AvgInten).

### Mitochondrial membrane potential (MMP) and superoxide detection

The mitochondrial changes of the APPsw cells were monitored using the fluorescent dyes, Rh123 and MitoSOX Red. The former is a cell permeable cationic dye that preferentially partitions into mitochondria based on the highly negative MMP. Depolarization of the MMP leads to the loss of Rh123 from the mitochondrion and appears an increased intracellular fluorescence [33]. The latter is a mitochondrial superoxide indicator, which is chemically targeted to mitochondria and exhibits red fluorescence when oxidized by superoxide [34]. Rh123 (Dojindo Laboratory, Kumamoto, Japan) and MitoSOX Red (Invitrogen, Carlsbad, CA, USA) were added to cell cultures to achieve a final concentration of 10 μM and 5 μM, respectively, for 30 min at 37°C after the APPsw cells were treated with pinocembrin plus copper as described above. The nucleic acid dye Hoechst 33342 (Dojindo Laboratory, Kumamoto, Japan) was used to identify the nucleus, and was added at a final concentration of 10 μM 10 min before the Rh123 and MitoSOX Red coincubation ended. Fluorescent images and intensities were acquired and analyzed by a Cellomics ArrayScan V^TI ^HCS Reader (Thermo Fisher Scientific Cellomics, Pittsburgh, PA, USA) combined with the Cell Health Profiling BioApplication Guide provided with the BioApplication software. Hoechst 33342, Rh123 and MitoSOX Red stained images were acquired using the 386/23 nm excitation and 460/40 nm emission, 485/20 nm excitation and 535/50 nm emission, and 549/15 nm excitation and 590/50 nm emission filters, respectively.

### RAGE expression analyses

The expression of RAGE in APPsw cells was determined by quantitative real-time PCR and immunofluorescence assay. After the APPsw cells were treated with pinocembrin plus copper as described above, total RNA was extracted. The human RAGE primer and probe consisted of forward primer, 5'-GATCCCCGTCCCACCTTCT-3'; reverse primer, 5'-GCTACTGCTCCACCTTCTG-3' (Genbank: NM_001206966.1).

RAGE protein expression was examined by immunofluorescence assay. Briefly, cells were fixed with 4% paraformaldehyde, permeabilized with 0.3% Triton X-100 (Sigma, St Louis, MO, USA), and then blocked with 3% bovine serum albumin (BSA; Sigma, St Louis, MO, USA) at room temperature. Next, cells were incubated with primary anti-RAGE antibody, followed by Alexa Fluor 488 conjugated secondary antibody (Invitrogen, Carlsbad, CA, USA). Fluorescent images and intensity were acquired and quantified by a Cellomics ArrayScan V^TI ^HCS Reader (Thermo Fisher Scientific Cellomics, Pittsburgh, PA, USA) and the Compartmental Analysis BioApplication Software Module. The optical fields were scanned with a 20 × objective lens to obtain a minimum of 1,000 cells per well. Mean average fluorescent intensity (Mean_AvgInten) was acquired and calculated as the value of RAGE protein expression.

### MAPK signal pathways, NFκB activation and apoptotic pathway assays

The MAPK signal pathways, NFκB activation and apoptotic pathway were all detected by immunofluorescence assay and quantified on the Cellomics ArrayScan V^TI ^high-content analysis platform, using fluorescence-based multiparametric technologies to monitor cellular constituent activities in fixed cells.

The APPsw cells were subcultured in black-walled optically clear-bottomed 96-well plates (Corning Life Sciences, Acton, MA, USA). After being treated with copper plus pinocembrin as described above, cells were fixed with 4% paraformaldehyde (Beijing Chemical Works, Beijing, China), permeabilized with 0.3% Triton X-100 (Sigma, St. Louis, MO, USA), and then blocked with 3% BSA (Sigma, St. Louis, MO, USA). The primary antibody mixture containing anti-phospho-p38 (Cell Signaling Technology, Bervely, MA, USA), anti-phospho-MAPKAP kinase-2 (MK2; Cell Signaling Technology, Bervely, MA, USA), anti-phospho-heat shock protein 27 (HSP27; Cell Signaling Technology, Bervely, MA, USA), anti-phospho-SAPK/JNK (Cell Signaling Technology, Bervely, MA, USA), anti-phospho-c-Jun (Cell Signaling Technology, Bervely, MA, USA), anti-phospho-p44/42 MAPK (Cell Signaling Technology, Bervely, MA, USA), anti-NFκB p65 (Cell Signaling Technology, Bervely, MA, USA), anti-B cell lymphoma 2 (Bcl-2; Cell Signaling Technology, Bervely, MA, USA), or anti-cytochrome *c *(Santa Cruz Biotechnology, Santa Cruz, CA, USA) in phosphate buffered saline (PBS, pH 7.4, 137 mM NaCl, 2.7 mM KCl, 10 mM Na_2_HPO_4 _· 2 H_2_O, 2 mM KH_2_PO_4_) was incubated for 2 h at room temperature. Subsequently, cells were incubated with corresponding secondary antibodies for 1 h. The primary antibodies and corresponding secondary antibodies are listed in Table 1.

**Table 1:** Primary antibodies and secondary antibodies used in this study

Primary antibody	Dilution	Source	Secondary antibody (dilution, source)
Phospho-p38 (Thr180/Tyr182) rabbit mAb	1:800	Cell Signaling Technology (CST)	Alexa Fluor 488 donkey anti-rabbit (1:500, Invitrogen, Carlsbad, CA, USA)
Phospho-MK2 (Thr334) rabbit mAb	1:200	CST	Alexa Fluor 488 donkey anti-rabbit (1:500, Invitrogen, Carlsbad, CA, USA)
Phospho-HSP27 (Ser82) rabbit mAb	1:50	CST	Alexa Fluor 488 donkey anti-rabbit (1:500, Invitrogen, Carlsbad, CA, USA)
Phospho-SAPK/JNK (Thr183/Tyr185) mouse mAb	1:400	CST	Alexa Fluor 488 donkey anti-mouse (1:500, Invitrogen, Carlsbad, CA, USA)
Phospho-c-Jun (Ser73) mouse mAb	1:100	CST	Alexa Fluor 555 donkey anti-mouse (1:500, Invitrogen, Carlsbad, CA, USA)
Phospho-p44/42 MAPK (Thr202/Tyr204) mouse mAb	1:200	CST	Alexa Fluor 488 donkey anti-mouse (1:500, Invitrogen, Carlsbad, CA, USA)
Anti-NFκB p65 rabbit mAb	1:200	CST	Alexa Fluor 488 donkey anti-rabbit (1:500, Invitrogen, Carlsbad, CA, USA)
Anti-Bcl-2 rabbit mAb	1:200	CST	Alexa Fluor 488 donkey anti-rabbit (1:500, Invitrogen, Carlsbad, CA, USA)
Anti-cytochrome *c *mouse mAb	1:400	Santa Cruz Biotechnology	Alexa Fluor 550 donkey anti-mouse (1:500, Invitrogen, Carlsbad, CA, USA)

High content analysis was performed on the Cellomics ArrayScan V^TI ^HCS Reader (Thermo Fisher Scientific Cellomics, Pittsburgh, PA, USA) using the Cytoplasm to Nucleus Translocation BioApplication [35,36]. Briefly, images were acquired via one to three independent channels with fixed exposure times. Based on the Hoechst nuclear stain in channel 1, a nuclear region mask was created and used to quantify nuclear protein distribution in the other target channels. By expanding the nuclear region mask, while remaining within cell boundaries, a concentric ring was generated and used as an approximation of the cytosolic compartment. For HSP27 and apoptotic detection, cytosolic fluorescent intensity was acquired and calculated as the value of protein expression. Nuclear intensity was monitored and used for evaluating c-Jun activation. Cytosolic and nuclear staining intensities were normalized to the total nuclear region and cytosolic ring area; this allows for the quantification of protein translocation between the nucleus and cytosol for p38, MK2, SAPK/JNK and NFκB p65. The capacity of translocation of the four proteins was illustrated by the value of Mean_CircRingAvgIntenDiff. Following scan completion, all data were exported to an Excel^® ^(Microsoft, Redmond, WA, USA) spreadsheet using the Cellomics vHCS View software and expressed as mean ± SEM.

### Caspase 3 and caspase 9 activity assay

Measurement of activity of caspase 3 and caspase 9 (Sigma, St. Louis, MO, USA) in the APPsw cells was performed using the caspase 3 and caspase 9 assay kits. Briefly, reactions were carried out in extraction buffer containing 200 μg of cytosolic protein extract and 40 μM Ac-DEVD p-nitroaniline or 40 μM Ac-LEHD p-nitroaniline. The reaction mixtures were incubated at room temperature for 2 h, and the formation of p-nitroaniline was measured at 405 nm. The concentration of p-nitroaniline released from the substrate was calculated from the absorbance values.

### Analysis of Aβ_1-42 _and tumor necrosis factor α (TNFα) by enzyme-linked immunosorbent assays (ELISAs)

Contents of Aβ_1-42 _and TNFα in culture medium of APPsw cells were measured by ELISA assays. Quantitative levels of Aβ and TNFα were measured according to the manufacturer's instructions (Jiameinuosi Biotech, Beijing, China). The optical density was measured at 450 nm, and values qualified from a standard curve generated with the limits of detection of 5 pg/ml for Aβ_1-42 _and 7 pg/ml for TNFα.

### Statistics

All data are represented as the mean ± SEM. Statistical significance was set at *P *< 0.05. Statistical analyses were performed on computer using the SPSS software (Version 13.0; SPSS, Inc., Chicago, IL, USA). Treatment differences in the escape latency in the MWM task were analyzed using two-factor analysis of variance with repeated measures on one factor. Tukey's *post hoc *test was used if the treatment and/or the treatment × day interaction were significant on analysis of variance (ANOVA). The other studies were analyzed using one-way ANOVA followed by an appropriate *post hoc *test to analyze the difference. Each of the *in vitro *experiments was repeated at least three times.

## Results

### Pinocembrin inhibited RAGE-induced p38MAPK and SAPK/JNK pathways of APPsw cells, not p44/42 MAPK in the presence of copper

#### Oral pinocembrin treatment ameliorated the spatial learning and memory deficits against A**β**_25-35_-induced toxicity

Spatial learning was initiated on the third day of pinocembrin treatment (day 1 for MWM test) and assessed by the time required to find the hidden platform (escape latency). Figure 1B shows the results of all mice during acquisition training. Repeated-measures ANOVA revealed a significant day effect on escape latency (*F*_(4,176) _= 8.39, *P *< 0.001) within the groups, indicating that all pinocembrin-treated mice improved their spatial learning effectively across the 5-day training period. There was also a significant treatment effect (*F*_(3,44) _= 16.68; *P *< 0.001) on the escape latency, and subsequent comparisons further suggested that 20 mg/kg and 40 mg/kg pinocembrin treatment reduced the escape latency in comparison to the Aβ_25-35 _group (20 mg/kg, *P*< 0.01; 40 mg/kg pinocembrin, *P*< 0.001) during the 5-day acquisition training, which demonstrated that pinocembrin was effective in attenuating spatial learning deficits in Aβ_25-35_-treated mice. There was no significance in the interactions of treatment and days (*F*_(12,176) _= 0.85, *P *> 0.05).

Furthermore, we investigated the effects of pinocembrin on spatial memory deficits. Probe trials were conducted on the ninth day of pinocembrin treatment (day 6 for MWM test) to assess the spatial memory. The length of time the mouse stayed in the target quadrant and the numbers of crossings where the platform had been located were both recorded. As shown in Figure 1C,D, the Aβ_25-35_-treated mice spent significantly less time searching for the platform in the target quadrant and showed less numbers of crossings where the platform had been previously relative to the sham mice (*P *< 0.001, *P *< 0.001). However, pinocembrin-treated mice spent more time searching in the target quadrant as compared to the Aβ_25-35_-treated mice (*P *< 0.01, *P *< 0.001). Pinocembrin treatment at 40 mg/kg also increased the numbers of crossings where the platform was located (*P *< 0.01). These results demonstrated that pinocembrin improved spatial memory against Aβ_25-35_-induced toxicity.

#### Oral pinocembrin treatment protected cerebral cortex ultrastructure against A**β**_25-35_-induced toxicity

**Figure 2:**
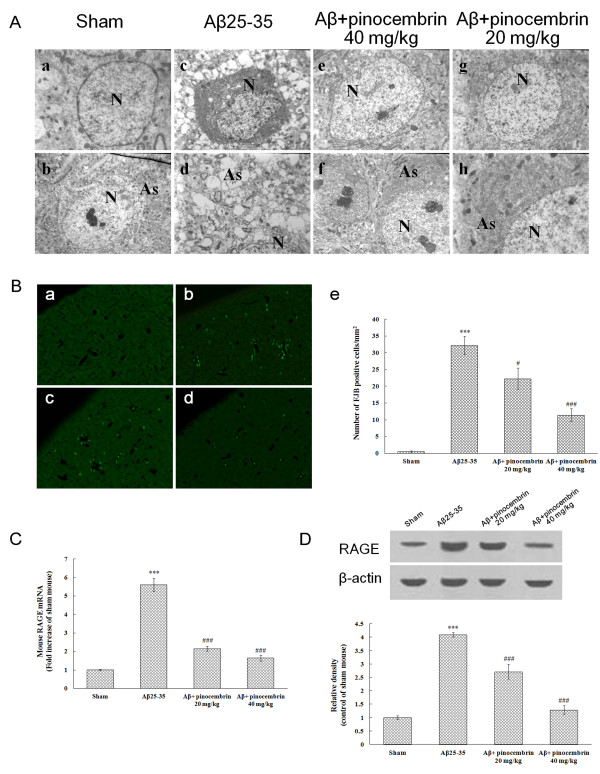
**Pinocembrin protected cerebral cortex ultrastructure and inhibited receptor for advanced glycation end products (RAGE) expression against amyloid-β peptide (Aβ)_25-35_-induced toxicity**. **(A) **Ultrastructural analysis of cerebral cortex against Aβ_25-35_-induced toxicity. (a,b) The representative area of sham mice cortex was characterized by the normal appearance of neurons, surrounding astrocytes and neuropil. Neurons (N) showed neither swelling nor shrinkage. Astrocytes (As) did not exhibit any apparent signs of perivascular edema. The neuropil appeared compact. (c,d) In the cortex of Aβ_25-35_-treated mice, neurons (N) showed degenerative changes. The cell membrane was ruptured, and the cell cytoplasm showed dark and dense granules. The nucleolus showed degenerative changes and the karyoplasm was filled with dense materials. Astrocyte (As) foot surrounding the neuron appeared swollen, producing an area of lower tissue density. (e,f) The 40 mg/kg pinocembrin treatment markedly attenuated damage of neuropil in the cortex. Neuronal pycnosis was relieved. The swelling of astrocytes (As) and disintegration of astrocytic cytoplasm was less apparent. (g,h) The 20 mg/kg pinocembrin treatment also alleviated neuronal pycnosis, and the neuropil in the treatment group appeared compact. **(B) **Fluoro-Jade B staining assay for cortical neuronal degeneration in Aβ_25-35_-infused mice. (a), sham; (b), Aβ_25-35_; (c), 20 mg/kg pinocembrin treatment; (d), 40 mg/kg pinocembrin treatment (× 100); (e), mean number of Fluoro-Jade B positive cells/mm^2 ^of the cerebral cortex. Data are presented as the mean ± SEM, n = 4, ***P < 0.001 vs sham, ^#^*P *< 0.05, ^###^*P *< 0.001 vs Aβ_25-35_. **(C) **Quantitative real-time polymerase chain reaction (PCR) for mouse RAGE mRNA in cerebral cortex in Aβ_25-35_-infused mice. Data are presented as the mean ± SEM, n = 5, ***P < 0.001 vs sham, ^###^*P *< 0.001 vs Aβ_25-35_. **(D) **Western blot analysis for mouse RAGE protein in cerebral cortex in Aβ_25-35_-infused mice. Data are presented as the mean ± SEM, n = 5, ***P < 0.001 vs sham, ^###^*P *< 0.001 vs Aβ_25-35_.

To further observe the morphological changes of the cerebral cortex, selected cortex pieces were viewed under a transmission electron microscope. The cerebral cortices of sham mice were characterized by the normal appearance of neurons and surrounding astrocytes (Figure 2A, a,b). Neurons (N) showed neither swelling nor shrinkage (Figure 2A, a). Astrocytes (As) did not exhibit any apparent signs of perivascular edema (Figure 2A, b). The neuropil appeared compact. In the cortices of Aβ_25-35_-treated mice, neuronal degeneration in the cortex was observed (Figure 2A, c,d). The cell membranes of neurons were ruptured, and the cell cytoplasm showed dark and dense granules (Figure 2A, c). The nucleolus showed degenerative changes and the karyoplasms were filled with dense materials (Figure 2A, c). Intracellular vacuolation and edema of astrocytes around neurons as well as adjacent degeneration of the neuropil were clearly visible (Figure 2A, d). Pinocembrin treatment at 20 mg/kg and 40 mg/kg markedly attenuated neuropil damage in the cortex. Neuronal pycnosis was relieved (Figure 2A, e,g). The swelling of As and disintegration of the astrocytic cytoplasm were less apparent (Figure 2A, f,h). Signs of membrane damage and vacuolation were less apparent.

#### Oral pinocembrin treatment inhibited neuronal degeneration in cerebral cortex against A**β**_25-35_-induced toxicity

FJB is a very useful maker for neuronal degeneration [29]. In the sham group, FJB-positive neurons were hardly observed in the cerebral cortex (Figure 2B, a). However, FJB-positive neurons were dramatically increased in the cerebral cortex of the Aβ_25-35 _group (*P *< 0.001, Figure 2B, b,e). Pinocembrin treatment significantly inhibited neuronal degeneration in the cerebral cortex, showing a decreased number of FJB-positive neurons in the pinocembrin-treated groups (*P *< 0.05, *P *< 0.001, Figure 2B, c-e). This result suggests that pinocembrin was effective in inhibiting the Aβ_25-35_-induced neuronal degeneration.

#### Oral pinocembrin treatment inhibit RAGE expression in cerebral cortex against A**β**_25-35_-induced toxicity

To assess the RAGE changes in the cerebral cortex, transcription and protein expression were studied following behavioral tests. An increased fold level of RAGE transcripts was observed in the cerebral cortex of Aβ_25-35_-treated mice (*P *< 0.001, Figure 2C). Western blot studies displayed increased RAGE protein expression in the cerebral cortex (*P *< 0.001, Figure 2D), which was consistent with the changes of the level of RAGE transcripts. Pinocembrin treatment significantly inhibited the upregulation of RAGE transcripts in accordance with its protein expression in a dose-dependent manner (*P *< 0.001, Figure 2C,D), suggesting that pinocembrin was effective in inhibiting the overexpression of RAGE in Aβ_25-35_-treated mice.

### Pinocembrin increased cell viability, but did not attenuate Aβ_1-42 _secretion and scavenge intracellular ROS of APPsw cells in the presence of copper

**Figure 3:**
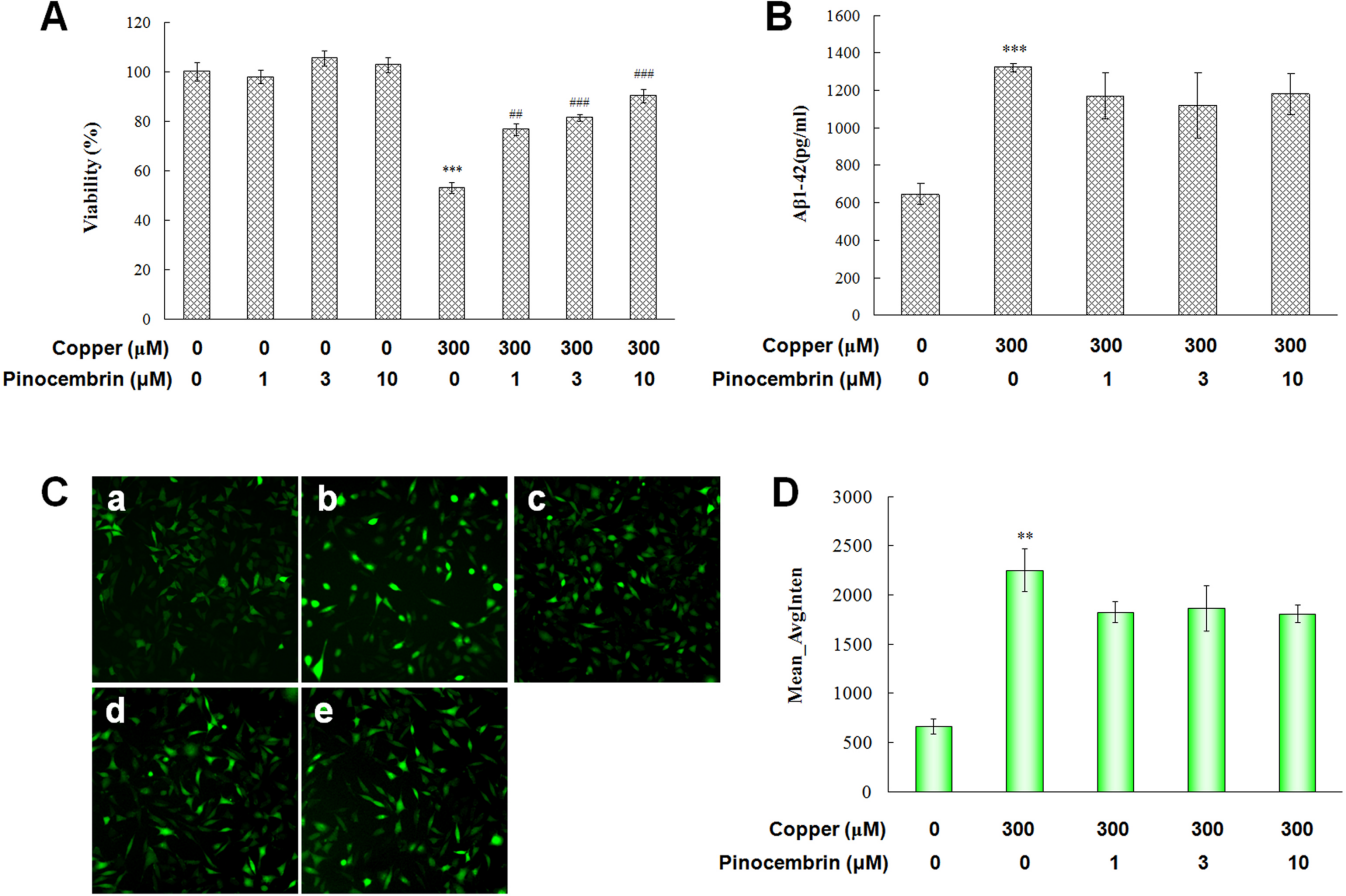
**Pinocembrin restored cell viability, but did not inhibit amyloid-β peptide (Aβ)_1-42 _secretion and scavenge intracellular reactive oxygen species (ROS) of human neuroblastoma SH-SY5Y cells overexpressing the Swedish mutant form of human APP (APPsw) in the presence of copper**. **(A) **Neuroprotective effects of pinocembrin evaluated by MTS (3-(4,5-dimethylthiazol-2-yl)-5-(3-carboxymethoxyphenyl)- 2-(4-sulfophenyl)-2H-tetrazolium, inner salt) assay. Data are expressed as mean ± SEM, n = 6, ***P < 0.001 vs control, ^##^*P *< 0.01, ^###^*P *< 0.001 vs copper. **(B) **Effects of pinocembrin on Aβ_1-42 _secretion of APPsw cells in the presence of copper. Data are expressed as mean ± SEM, n = 6, ***P < 0.001 vs control. **(C,D) **Effects of pinocembrin on intracellular ROS generation in APPsw cells in the presence of copper. Intracellular ROS levels were determined based on the 2',7'-dichlorofluorescein (DCF) fluorescence on the ArrayScan high-content screening (HCS) Reader with the Morphology Explorer BioApplication. (a), APPsw group; (b), APPsw cells in the presence of 300 μM copper; (c), APPsw cells in the present of 300 μM copper with pinocembrin treatment at 1.0 μM; (d), 3.0 μM; (e), 10.0 μM. Data are expressed as mean ± SEM, n = 6, **P < 0.01 vs control.

In order to better resemble the *in vivo *Aβ-induced neurotoxicity, instead of directly using single Aβ treatment, we used a copper-treated APPsw overexpressing cell system [32]. As shown in Figure 3A, cell viability was significantly decreased in the presence of 300 μM copper in APPsw cells (*P *< 0.001). Pinocembrin enhanced the cell viability at 1.0 μM, 3.0 μM and 10.0 μM in a dose-dependent manner (*P *< 0.01, *P *< 0.001, *P *< 0.001). Pinocembrin did not show a significant effect in the APPsw cells without copper treatment for 24 h.

Copper is one of the redox metals, capable of increasing oxidative stress with the production of excess superoxide and hydroxyl radicals due to the overproduction of Aβ [37], and thus is associated with the severe redox imbalance in this cell model [32]. Copper increased the Aβ_1-42 _and ROS generation by about a 4.2-fold and 3.3-fold increase, respectively (*P *< 0.01, Figure 3B-D). However, pinocembrin could not significantly inhibit Aβ_1-42 _secretion and scavenge the ROS generation in the present model at the each concentration, which indicated that pinocembrin did not have a sufficient effect on decreasing Aβ_1-42 _secretion and ameliorating the antioxidative ability in APPsw cells subjected to Aβ-induced neurotoxicity triggered by copper.

### Pinocembrin inhibited RAGE expression both in RAGE-overexpression cells and APPsw cells in the presence of copper

To further verify the expression of RAGE and the effect of pinocembrin, we used an Aβ_1-42_-treated RAGE overexpressing cell model, where the RAGE expression could be elevated by excessive amounts of Aβ. Meanwhile, we also detected the levels of RAGE mRNA and protein in an AD cell model by using copper to trigger the neurotoxicity of Aβ.

**Figure 4:**
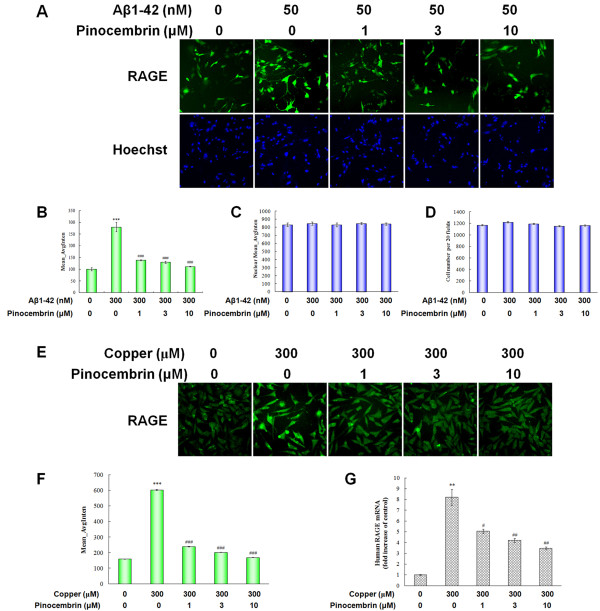
**Pinocembrin inhibited receptor for advanced glycation end products (RAGE) expression in RAGE-overexpression cells and in human neuroblastoma SH-SY5Y cells overexpressing the Swedish mutant form of human APP (APPsw) in the presence of copper**. **(A) **Effects of pinocembrin on RAGE expression in RAGE-overexpression cells. **(B) **Mean_AvgInten value of enhanced green fluorescent protein (EGFP)-RAGE in RAGE-overexpression cells being exposed to amyloid-β peptide (Aβ)_1-42 _for 24 h. Inhibition of RAGE expression results in changes of Mean_AvgInten value of EGFP-RAGE. Data are expressed as mean ± SEM, n = 6, ***P < 0.001 vs control, ^###^*P *< 0.001 vs Aβ_1-42_. **(C) **Nuclear Mean_AvgInten value in RAGE-overexpression cells cocultured with pinocembrin for 24 h. Data are expressed as mean ± SEM, n = 6. **(D) **Cell numbers per 20 fields of RAGE-overexpression cells cocultured with pinocembrin for 24 h. Data are expressed as mean ± SEM, n = 6. **(E) **Effects of pinocembrin on RAGE expression in APPsw cells in the presence of copper. **(F) **Mean_AvgInten value of EGFP-RAGE in RAGE-overexpression cells being exposed to Aβ_1-42 _for 24 h. Inhibition of RAGE expression results in changes of Mean_AvgInten value of EGFP-RAGE. Data are expressed as mean ± SEM, n = 6, ***P < 0.001 vs control, ^###^*P *< 0.001 vs copper. **(G) **Quantitative real-time polymerase chain reaction (PCR) for human RAGE mRNA in APPsw cells in the presence of copper. Data are presented as the mean ± SEM, n = 6, **P < 0.01 vs control, ^#^*P *< 0.05, ^##^*P *< 0.01 vs copper.

As shown in Figure 4A,B, Aβ_1-42 _triggered the upregulation of RAGE by about a 3.77-fold increase in Mean_AvgInten values (*P *< 0.001) in the present model. Pinocembrin inhibited the overexpression of RAGE (*P *< 0.001). The inhibition values of pinocembrin are 59.66% ± 2.06%, 65.89% ± 1.46% and 71.51% ± 0.98% at the concentrations of 1.0 μM, 3.0 μM and 10.0 μM, respectively. In addition, Aβ_1-42 _induced RAGE transcription without any cell viability influence due to non-significant changes on nuclear number and fluorescent intensity in RAGE-expressing cells for 24 h, and pinocembrin did not show significant effects on RAGE-expressing cell numbers in the same manner (Figure 4C,D). This phenomenon might suggest that pinocembrin has a direct inhibitory effect on RAGE expression, probably not dependent on the proliferation of the cells.

Immunofluorescence assays and quantitative real-time PCR showed a 2.8-fold increase in Mean_AvgInten values of RAGE expression and an eightfold increase level of RAGE transcripts in copper-treated APPsw cells (*P *< 0.001, *P *< 0.01, Figure 4E-G). Pinocembrin treatments significantly inhibited RAGE protein expression in accordance with the downregulation of the transcripts at concentrations of 1.0 μM, 3.0 μM and 10.0 μM in a dose-dependent manner (*P *< 0.05, *P *< 0.01, *P *< 0.001, Figure 4E-G), suggesting that pinocembrin was prominent in inhibiting the overexpression of RAGE in copper-treated APPsw cells.

### Pinocembrin inhibited RAGE-induced p38MAPK and SAPK/JNK pathways of APPsw cells, not p44/42 MAPK in the presence of copper

**Figure 5:**
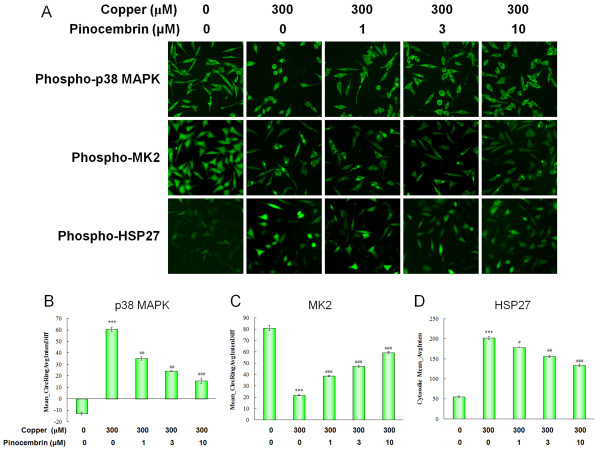
**Pinocembrin inhibited receptor for advanced glycation end products (RAGE)-induced p38 mitogen-activated protein kinase (MAPK)-MAPKAP kinase-2 (MK2)-heat shock protein 27 (HSP27) pathway of human neuroblastoma SH-SY5Y cells overexpressing the Swedish mutant form of human APP (APPsw) in the presence of copper**. **(A) **Images of p38MAPK-MK2-HSP27 pathways performed by high content analysis on the ArrayScan high-content screening (HCS) Reader using the Cytoplasm to Nucleus Translocation BioApplication. APPsw cells were treated plus copper as described in the Methods, prior to fixation, permeabilization, Hoechst nuclear staining, and immunolabeling with antibodies targeting phospho-p38MAPK, phospho-MK2, and phospho-HSP27. Each column reflects images collected from the respective fluorescent channels. **(B,C) **Values of Mean_CircRingAvgIntenDiff describing the capacity translocation of cytosolic phospho-p38 to the nucleus and nuclear phospho-MK2 to the cytoplasm. **(D) **Cytosolic Mean_AvgInten value illustrating the expression of phospho-HSP27. Data are expressed as mean ± SEM, n = 6, ***P < 0.001 vs control, ^#^*P *< 0.05, ^##^*P *< 0.01, ^###^*P *< 0.001 vs copper.

Aβ-RAGE signaling is a mediator in the phosphorylation state of MAPKs. Total p38MAPK cellular distribution and expression levels are thought to modulate the downstream highly expressed substrate MK2, and subsequently HSP27. In control cells, basal levels of phospho-p38 and phospho-MK2 were significantly confined to the cytosolic and nuclear compartment, showing negative and high Mean_CircRingAvgIntenDiff values, respectively. The downstream phospho-HSP27 showed correspondingly weak staining, showing a low cytosolic Mean_AvgInten value. In copper-treated APPsw cells, the capacity of copper to promote phospho-p38 and phospho-MK2 translocation was illustrated by a significant increase and a remarkable decrease in Mean_CircRingAvgIntenDiff values, respectively (*P *< 0.001, *P *< 0.01, Figure 5A-C). Similarly, the downstream phospho-HSP27 showed strong fluorescence with a 3.74-fold increase in cytosolic Mean_AvgInten values (*P *< 0.001, Figure 5A,D). Pinocembrin treatments significantly inhibited the p38MAPK signal pathway. The translocation of cytosolic phospho-p38 to the nucleus and nuclear phospho-MK2 to the cytoplasm were significantly inhibited in accordance with the downregulation of downstream phospho-HSP27 at the concentrations of 1.0 μM, 3.0 μM and 10.0 μM in a dose-dependent manner (*P *< 0.05, *P *< 0.01, *P *< 0.001, Figure 5).

**Figure 6:**
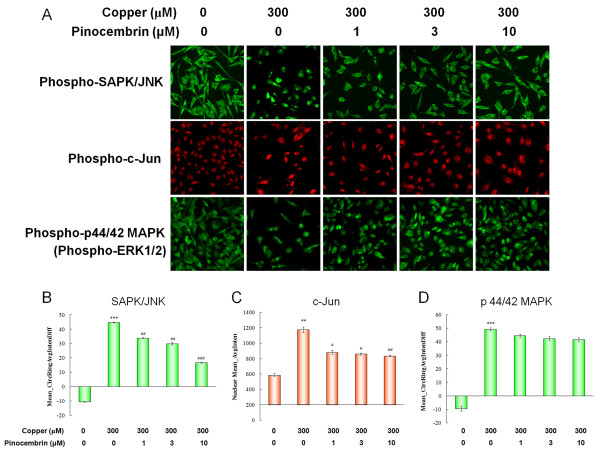
**Pinocembrin inhibited receptor for advanced glycation end products (RAGE)-induced stress-activated protein kinase (SAPK)/c-Jun N-terminal kinase (JNK) pathway, but not p44/42 mitogen-activated protein kinase (MAPK) pathway of human neuroblastoma SH-SY5Y cells overexpressing the Swedish mutant form of human APP (APPsw) in the presence of copper**. **(A) **Images of SAPK/JNK and p44/42 MAPK pathways evaluated by high content analysis on the ArrayScan high-content screening (HCS) Reader using the Cytoplasm to Nucleus Translocation BioApplication. **(B,D) **Values of Mean_CircRingAvgIntenDiff describing the capacity translocation of cytosolic phospho-JNK and phospho-p44/42 MAPK to the nucleus. **(C) **Nuclear Mean_AvgInten value illustrating the expression of phospho-c-Jun. Data are expressed as mean ± SEM, n = 6, **P < 0.01, ***P < 0.001 vs control, ^#^*P *< 0.05, ^##^*P *< 0.01, ^###^*P *< 0.001 vs copper.

As a consequence of Aβ-RAGE interaction, activation of SAPK/JNK and p44/42 MAPK pathways was also observed. The basal level of phospho-SAPK/JNK and phospho-p44/42 MAPK was both confined to the cytosolic compartment, shown as negative Mean_CircRingAvgIntenDiff values in control cells. In copper-treated cells, phospho-SAPK/JNK and phospho-p44/42 MAPK translocation was promoted by a significant increase in Mean_CircRingAvgIntenDiff values, respectively (*P *< 0.001, Figure 6A,B,D). The level of phospho-c-Jun was shown by consistent changes in Mean_AvgInten values in the nucleus (*P *< 0.01, Figure 6A,C). Pinocembrin treatments significantly inhibited the SAPK/JNK pathway. The translocation of cytosolic phospho-SAPK/JNK to the nucleus and the downregulation of its downstream phospho-c-Jun were both inhibited at the concentrations of 1.0 μM, 3.0 μM and 10.0 μM in a dose-dependent manner (*P *< 0.05, *P *< 0.01, *P *< 0.001, Figure 6A-C). However pinocembrin did not show a significant effect on phospho-p44/42 MAPK nuclear translocation.

### Pinocembrin inhibited the NFκB p65 translocation and the release of inflammatory cytokines in APPsw cells in the presence of copper

**Figure 7:**
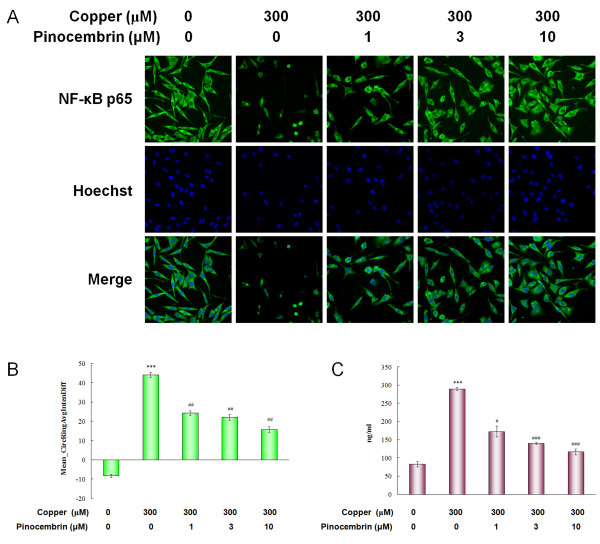
**Pinocembrin inhibited the nuclear factor κB (NFκB) p65 translocation and the release of inflammatory cytokines of human neuroblastoma SH-SY5Y cells overexpressing the Swedish mutant form of human APP (APPsw) in the presence of copper**. **(A) **Images of NFκB p65 translocation in the same field. NFκB activity was analyzed on the ArrayScan high-content screening (HCS) Reader to quantify the translocation of cytosolic p65 to the nucleus. **(B) **The value of Mean_CircRingAvgIntenDiff describing the translocation capacity of cytosolic p65 to the nucleus. Data are expressed as mean ± SEM, n = 6, ***P < 0.001 vs control, ^##^*P *< 0.01 vs copper. **(C) **Content of tumor necrosis factor α (TNFα) in APPsw cell culture supernatant after being exposed to copper. Data are expressed as mean ± SEM, n = 4, ***P < 0.001 vs control, ^#^*P *< 0.05, ^###^*P *< 0.001 vs copper.

NFκB activity was analyzed by quantifying the translocation of cytosolic p65 to the nucleus. As shown in Figure 7A,B, basal p65 was mainly distributed in the cytoplasm, and Mean_CircRingAvgIntenDiff values were negative in control cells. The p65 translocation from the cytoplasm to the nucleus was caused in the copper-treated cells. Mean_CircRingAvgIntenDiff values drastically increased from -8.21 ± 0.85 in control cells to 44.14 ± 1.21 in copper-treated cells (*P *< 0.001, Figure 7A,B). This translocation was significantly inhibited by pinocembrin treatment. The translocation of p65 to the nucleus was inhibited at the concentrations of 1.0 μM, 3.0 μM and 10.0 μM in a dose-dependent manner (*P *< 0.01, *P *< 0.01, *P *< 0.01, Figure 7A,B).

The values of TNFα in APPsw cell culture supernatant changed in the same way. APPsw cells increased the generation of TNFα following copper treatment, but the secretions were significantly attenuated by pinocembrin at 1.0 μM, 3.0 μM and 10.0 μM in a dose-dependent manner (*P *< 0.05, *P *< 0.001, *P *< 0.001, Figure 7C).

### Pinocembrin protected mitochondrial function and inhibited mitochondrion-induced apoptosis

**Figure 8:**
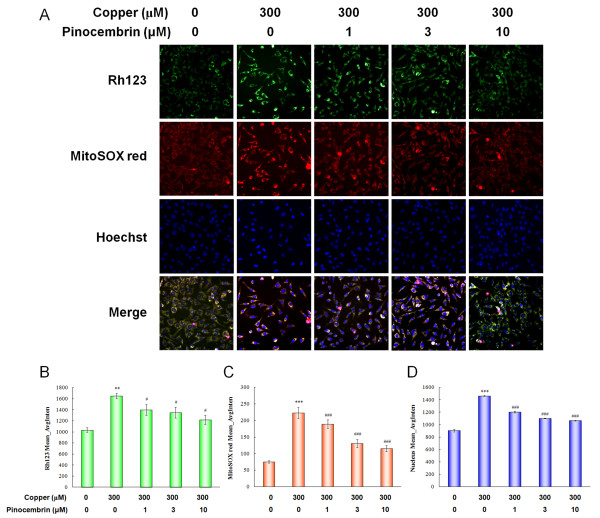
**Pinocembrin protected mitochondrial function and inhibited nuclear apoptosis**. **(A) **Fluorescent images of mitochondrial function and nuclear changes in the same field. The mitochondria were monitored using the fluorescent dyes, Rh123 and MitoSOX Red, in living cells. Fluorescent images and intensities were acquired and analyzed by a Cellomics ArrayScan high-content screening (HCS) Reader combined with the Cell Health Profiling BioApplication Guide provided with the BioApplication software. **(B-D) **Values of Mean_AvgInten of Rh123, MitoSOX Red and Hoechst 33342 collected from the respective fluorescent channels using the same optical field. Data are expressed as mean ± SEM, n = 6, **P < 0.01, ***P < 0.001 vs control, ^#^*P *< 0.05, ^###^*P *< 0.001 vs copper.

The mitochondrial function of the APPsw cells was monitored using the fluorescent dyes Rh123 and MitoSOX Red. The fluorescence of Rh123 and MitoSOX Red were evaluated by Mean_AvgInten values collected from the respective fluorescent channels using the same optical field. After incubation with copper for 24 h, the Mean_AvgInten values of Rh123 and MitoSOX red increased to 59.61% and 196.17% above control cells, respectively (*P *< 0.01, *P *< 0.001, Figure 8A-C). These data represented a loss of MMP and a special oxidation of mitochondria. Pinocembrin alleviated mitochondrial dysfunction remarkably. Treatment at 1.0 μM, 3.0 μM and 10.0 μM reduced the respective Mean_AvgInten value in a dose-dependent manner (*P *< 0.05, *P *< 0.001, Figure 8A-C).

The cytoprotective effects of pinocembrin were also confirmed by Hoechst 33342 staining. As shown in Figure 8A,D, copper-treated APPsw cells contained condensed or fragmented nuclei with strong bright Hoechst staining (*P *< 0.001). However, pinocembrin attenuated nuclear condensation shown as lowered nuclear Mean_AvgInten values (*P *< 0.001).

**Figure 9:**
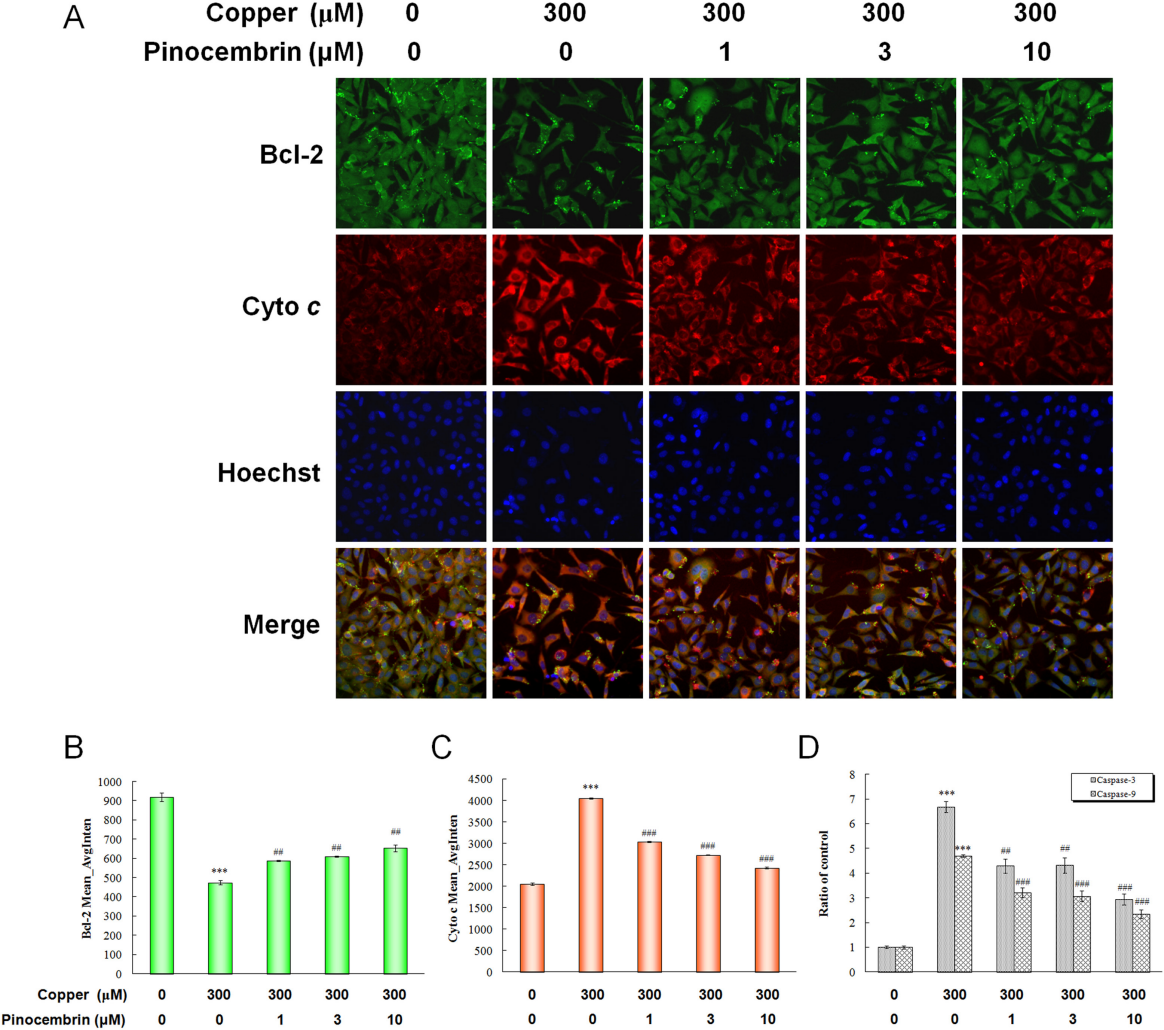
**Pinocembrin inhibited mitochondrion-induced apoptosis**. **(A) **Fluorescent images of B cell lymphoma 2 (Bcl-2), cytochrome *c *and nucleus in the same field. **(B,C) **Values of Mean_AvgInten of Bcl-2 and cytochrome *c *collected from the respective fluorescent channels using the same optical field. Data are expressed as mean ± SEM, n = 6, ***P < 0.001 vs control, ^##^*P *< 0.01, ^###^*P *< 0.001 vs copper. **(D) **Activity of caspase 3 and caspase 9 of human neuroblastoma SH-SY5Y cells overexpressing the Swedish mutant form of human APP (APPsw) in the presence of copper. Data are expressed as mean ± SEM, n = 4, ***P < 0.001 vs control, ^##^*P *< 0.01, ^###^*P *< 0.001 vs copper.

Mitochondrial dysfunction, caspase-dependent toxicity, and downstream signaling pathways are documented as critical apoptotic events during AD processes. The release of cytochrome *c*, the expression of Bcl-2, and the cytosolic activity of caspase 3 and caspase 9 were detected as mitochondrial related apoptotic molecular makers. The two formers were measured by immunofluorescence labeling by quantifying the Mean_AvgInten values in the same optical field. The Mean_AvgInten values of Bcl-2 saw a significant decrease, while the values of cytochrome *c *saw a two-fold increase (*P *< 0.001, Figure 9A-C). Pinocembrin caused the restoration of Bcl-2 and cytochrome *c *(*P *< 0.01, *P *< 0.001, Figure 9A-C). Similarly, the activities of caspase 3 and caspase 9 were both increased in APPsw cells in the presence of copper, but they were significantly suppressed by pinocembrin treatment (*P *< 0.01, *P *< 0.001, Figure 9D). These restorative effects were significant at the concentrations of 1.0 μM, 3.0 μM and 10.0 μM in a dose-dependent manner.

## Discussion

There are three major findings from the present study. First, pinocembrin, for the first time, is shown to be a promising drug candidate for the treatment of AD as it is shown to alleviate the cognitive deficits induced by Aβ_25-35 _intracerebroventricular infusion in mice *in vivo *and reduce neuronal damage and degeneration mediated by Aβ in the presence of copper *in vitro*. Second, the survival signaling pathways of the actual therapeutic value of pinocembrin have been demonstrated through inactivation of RAGE-dependent signaling pathways and inhibition of mitochondrion-mediated apoptosis against Aβ-mediated neurotoxicity. Third, RAGE was used as a therapeutic target for evaluation of the efficacy of compounds, and an effective inhibitory effect of pinocembrin was illustrated, as well as the subsequent inactivation of p38MAPK, SAPK/JNK pathways and the downstream inflammatory response.

The toxic effects of Aβ on the cholinergic system and cognitive function are variable, due to the differences in the sites of administration and experimental models [38-44]. The neurotoxicity of Aβ interrelated with senile plaques in AD brains is linked to the amino acids located in positions 25 to 35 of the full length protein [45,46]. In order to investigate the prospective therapeutic value of pinocembrin on cognitive impairment and neuronal apoptosis in correlation with neurodegeneration in AD, Aβ neurotoxicity induced learning and memory deficits and neuronal injury were established by a single intracerebroventricular infusion of Aβ_25-35_, an Aβ_1-42_-treated RAGE overexpressing cell model, and a copper-treated APPsw overexpressing cell system, all of which could well reflect Aβ toxicity in an Aβ-rich environment both *in vivo *and *in vitro*.

Intracerebroventricular infusion of Aβ_25-35 _was demonstrated to induce spatial learning and memory impairment in AD animal models [41-44]. Herein, a single intracerebroventricular injection of Aβ_25-35 _in mice induced significant amnesia as compared to the sterile saline -injected sham group. This confirmed that cognitive impairment was induced by Aβ_25-35 _peptide itself, and was not attributable to an intracerebroventricular injection. Our results showed that pinocembrin, taken by oral gavage of 20 mg/kg/day and 40 mg/kg/day, improved spatial learning effectively across the 5-day acquisition training period. The Aβ_25-35_-administered mice after pinocembrin treatment showed a better learning capability in finding the hidden platform by reduction of escape latency. In the memory probe trial, the Aβ_25-35_-treated mice receiving pinocembrin treatment performed much better in searching for the target quadrant and the site where the platform was located as compared to the Aβ_25-35_-administered mice.

Cerebral histomorphological or functional anomalies could be a sign of the pathogenesis of neurodegeneration, accompanied by cognitive disorder in AD patients [47]. Our study further confirmed that pinocembrin preserved the ultrastructural changes of neurons and surrounding astrocytes in the cerebral cortex as well. Both 20 mg/kg and 40 mg/kg pinocembrin treatment protected the neuropil from Aβ_25-35_-induced toxicity, characterized by the relatively normal appearance of neurons and surrounding astrocytes, without shrinkage or swelling. The immunohistochemical findings from the FJB staining assay were consistent with the ultrastructural results. The total number of FJB-positive cells in the cerebral cortex showed a significant reduction after pinocembrin treatment, indicating that pinocembrin might be effective in decreasing neurodegeneration and improving cerebral histomorphological outcomes. As a result of the current study on *in vivo *cognitive function, neuronal ultrastructural investigation and neuronal degeneration detection, pinocembrin has been shown to be potentially beneficial for the treatment of AD. Therefore, the underlying mechanism of its actual therapeutic value has been explored and is discussed as below.

Oxidative stress plays a critical role in AD pathogenesis. ROS production can be largely catalyzed by transition metals, such as copper [48,49]. The association of copper and Aβ toxicity is mainly suggested in three aspects: (1) the effect on cell viability correlated with Aβ; (2) Aβ-induced neurotoxicity relevant to oxidative stress indicated by ROS production; and (3) the effect of copper in Aβ aggregation. Here, we detected that copper increased ROS generation by about a 3.3-fold increase, in accordance with Aβ peptide secretion and decrease of cell viability. However, pinocembrin did not affect Aβ_1-42 _secretion. It modestly and non-significantly scavenged ROS generation. Pinocembrin belongs to the flavonoids, which are thought to be effective in quenching free radicals [50]. Although it showed the antioxidative activities in scavenging ROS production and decreasing some of oxidant enzyme activity in reducing ischemic injury [22,25], pinocembrin did not provide sufficient antioxidant effect through scavenging ROS generation against Aβ-mediated neurotoxicity stimulated by copper. Our present data suggest that pinocembrin may act synergistically with other mechanisms for the treatment of AD.

Evidence has indicated that APP localizes not only to the plasma membrane, but also to the mitochondrial membrane, trans-Golgi network, endoplasmic reticulum, and lysosomal membrane [51-53]. Therefore, two potential pathways may underlie the neurotoxicity of intraneuronal Aβ: (1) Aβ secreted into extracellular space is subsequently taken up by neurons at the neuronal cell surface, and causes neuronal dysfunction mainly via RAGE-dependent pathway; (2) Aβ produced intracellularly remains within the neuron, and promotes toxicity to various cytoplasmic organoids [54]. According to the possible neurotoxic pathways of Aβ, our following explanations for the therapeutic mechanism of pinocembrin consist of the above two aspects.

RAGE is a potential therapeutic target in Aβ-induced processes in AD neuropathology. Neuronal RAGE is highly overexpressed in AD patients, and has capacity to bind various forms of Aβ at the neuronal cell surface [12,55,56]. The neurotoxic response to Aβ-RAGE correlates with activation the three subfamilies of MAPKs, p38MAPK, SAPK/JNK, and extracellular signal-regulated kinase (ERK1/2, p44/42 MAPK), and NFκB transduction. Among these toxic transductions, p38-MK2-HSP27 and JNK/c-Jun have been shown to play important roles in Aβ/RAGE-induced synaptic dysfunction. These two pathways may be triggered by RAGE/Aβ as downstream signaling cascades that contribute to the early phases of AD [57-59].

**Figure 10:**
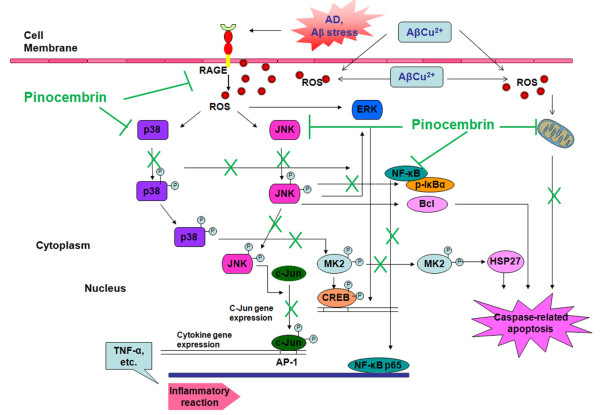
**Schematic diagram of the neuroprotective pathways of pinocembrin on amyloid-β peptide (Aβ)-induced neurotoxicity**.

Based on our *in vivo *and *in vitro *studies, one of the explanations for the molecular mechanism of pinocembrin is the regulation of Aβ/RAGE-mediated pathways (Figure 10). Neuronal RAGE transcripts and protein expression were significantly upregulated in Aβ-mediated neurotoxicity models, which is in good agreement with previous AD animal models [56,59]. As a consequence of RAGE overexpression and Aβ-RAGE activation, p38MAPK-MK2-HSP27 and SAPK/JNK-c-Jun cascades were phosphorylated and activated in the copper-treated APPsw overexpressing cell system. Pinocembrin significantly inhibited the upregulation of RAGE transcripts in accordance with its protein expression both *in vivo *and *in vitro*. In the Aβ_1-42_-treated RAGE overexpressing cell system, an evaluating *in vitro *model with a strong response region of wild-type human RAGE promoter for inducing RAGE expression in which Aβ acts as a stimulator when supplemented in culture medium, pinocembrin showed a direct inhibitory effect on RAGE expression. Thereby we confirmed that pinocembrin was effective in inhibiting the overexpression of RAGE against Aβ-mediated toxicity, and speculate that pinocembrin may affect RAGE transcription by interacting with the gene promoter, affecting the regulatory factors of the gene promoter, or stabilizing mRNAs in the mRNA processing or post-transcriptional regulation levels. Our results further revealed that pinocembrin markedly inhibited the activation of p38MAPK-MK2-HSP27 and SAPK/JNK-c-Jun pathways, accompanied by a weak and apparently ineffective inhibitory effect on ERK1/2 activation. These inhibitory effects of pinocembrin may be secondary to the lowering of the Aβ-RAGE interaction.

NFκB activation is also a neurotoxic signaling event subsequent to Aβ-RAGE activation. RAGE-mediated activation of NFκB by Aβ in the brain could lead either to comprehensive inflammation or to signaling that could result in apoptosis. In our *in vitro *study, pinocembrin significantly hampered NFκB p65 nuclear translocation and inhibited inflammatory factor release. Based on the present data and previous reports [22,60,61], the anti-inflammatory effect of pinocembrin demonstrated here might be either a direct effect independent of Aβ toxicity or a secondary effect subsequent to the Aβ-RAGE interaction (Figure 10).

Another explanation for pinocembrin promoting cognitive function and preserving neuronal ultrastructure against Aβ-induced toxicity is that pinocembrin protected mitochondria and regulated mitochondrion-mediated apoptosis (Figure 10). Mitochondria play a pivotal role in apoptosis signaling pathways. Loss of MMP contributes to cell death by reducing ATP production, increasing production of ROS, and enhancing release of deadly signal molecules from the intermembrane space, thereby leading to caspase-dependent toxicity and downstream apoptotic signaling [62]. As discussed above, Aβ also localizes to the mitochondrial membrane and has a direct toxicity to mitochondrial function. Further, activation of MAPKs signaling pathways in response to a variety of stressors such as oxidative stress, also leads to neuronal apoptosis via the mitochondria-dependent pathway [63]. According to our study, a loss of MMP and a special oxidation of mitochondria were apparent in APPsw cells in the presence of copper, coincidence with intracellular ROS overgeneration and MAPKs activation. As previous studies have reported, pinocembrin showed a direct protective effect on mitochondria [22,27], and we also observed that pinocembrin alleviated mitochondrial dysfunction through improving mitochondrial membrane potential and protecting mitochondria from oxidative stress.

Of the apoptotic molecular makers during the mitochondrion-dependent pathway, Bcl-2, localized to mitochondria, may prevent apoptotic events by lowering the amount of free Ca^2+ ^and increasing the tolerance of mitochondria to high calcium loads [64]. Once mitochondrial membrane permeabilization is induced, cytochrome *c *is released, forming an oligomeric complex with dATP and Apaf-1 [65], accompanied by recruitment of procaspase 9 and its activation. In this study, cytochrome *c*, Bcl-2 and caspase 3 and caspase 9 were all were changed in APPsw cells in the presence of copper. Pinocembrin inhibited mitochondrion-dependent apoptosis. Nuclear condensation was also attenuated by pinocembrin treatment.

Additionally, there are other molecular components involved in apoptosis from different upstream signaling pathways. The release of cytochrome *c *is tightly linked to the presence and activation of JNK, and the JNK mediated cytochrome *c *release contributing to caspase 3 activation and apoptosis onset [66,67]. In addition, HSP27 regulates apoptosis by inhibiting the release of cytochrome *c *and preventing the activation of caspase 3 [68]. Combined with this evidence, pinocembrin is also illustrated to modulate apoptosis synergistically with the inactivation of SAPK/JNK-c-Jun pathway.

## Conclusions

With the experimental design and methodology used in the present study, we investigated the therapeutic value of pinocembrin in cognitive function and neuronal protection against Aβ-induced toxicity. Furthermore, we explored the therapeutic targets for the efficacy of pinocembrin, and demonstrated the underlying mechanisms of pinocembrin through inhibiting RAGE-dependent signaling pathway and regulating mitochondrion-mediated apoptosis. In summary, pinocembrin appears to be a promising candidate for the prevention and therapy of Alzheimer's disease.

## Competing interests

The authors declare that they have no competing interests.

## Authors' contributions

RL participated in the design of the study, and carried out the animal experiments as well as the TEM analysis and drafted the manuscript. C-xW performed RAGE expression analyses and FJB staining assay. RL, DZ and ST carried out cell culture and gene transfection experiments. FY and LZ evaluated and analyzed the MAPK signal pathways, NFκB activation and apoptotic pathway transduction. T-tZ carried out mitochondrial assays. RL, DZ and T-tZ performed the statistical analysis. G-hD conceived of the study and participated in its design and coordination and helped to draft the manuscript. All authors read and approved the final manuscript.

## Pre-publication history

The pre-publication history for this paper can be accessed here:

http://www.biomedcentral.com/1741-7015/10/105/prepub
